# ^1H^NMR-Based metabolomic profiling method to develop plasma biomarkers for sensitivity to chronic heat stress in growing pigs

**DOI:** 10.1371/journal.pone.0188469

**Published:** 2017-11-27

**Authors:** Samir Dou, Nathalie Villa-Vialaneix, Laurence Liaubet, Yvon Billon, Mario Giorgi, Hélène Gilbert, Jean-Luc Gourdine, Juliette Riquet, David Renaudeau

**Affiliations:** 1 PEGASE, INRA, Agrocampus Ouest, St Gilles, France; 2 MIA-T, INRA, Université de Toulouse, INP, ENSAT, ENVT, Castanet Tolosan, France; 3 GenPhySE, INRA, Université de Toulouse, INP, ENSAT, ENVT, Castanet Tolosan, France; 4 PTEA, INRA, Petit-Bourg (Guadeloupe), France; 5 GenESI, INRA, Surgères, France; 6 URZ, INRA, Petit-Bourg (Guadeloupe), France; Wageningen UR Livestock Research, NETHERLANDS

## Abstract

The negative impact of heat stress (HS) on the production performances in pig faming is of particular concern. Novel diagnostic methods are needed to predict the robustness of pigs to HS. Our study aimed to assess the reliability of blood metabolome to predict the sensitivity to chronic HS of 10 F1 (Large White × Creole) sire families (SF) reared in temperate (TEMP) and in tropical (TROP) regions (n = 56±5 offsprings/region/SF). Live body weight (BW) and rectal temperature (RT) were recorded at 23 weeks of age. Average daily feed intake (AFDI) and average daily gain were calculated from weeks 11 to 23 of age, together with feed conversion ratio. Plasma blood metabolome profiles were obtained by Nuclear Magnetic Resonance spectroscopy (^1H^NMR) from blood samples collected at week 23 in TEMP. The sensitivity to hot climatic conditions of each SF was estimated by computing a composite index of sensitivity (I_*sens*_) derived from a linear combination of *t* statistics applied to familial BW, ADFI and RT in TEMP and TROP climates. A model of prediction of sensitivity was established with sparse Partial Least Square Discriminant Analysis (*s*PLS-DA) between the two most robust SF (n = 102) and the two most sensitive ones (n = 121) using individual metabolomic profiles measured in TEMP. The sPLS-DA selected 29 buckets that enabled 78% of prediction accuracy by cross-validation. On the basis of this training, we predicted the proportion of sensitive pigs within the 6 remaining families (n = 337). This proportion was defined as the predicted membership of families to the sensitive category. The positive correlation between this proportion and I_*sens*_ (r = 0.97, P < 0.01) suggests that plasma metabolome can be used to predict the sensitivity of pigs to hot climate.

## Introduction

The economic losses in pig industry due to heat stress (HS) are important both for tropical countries where the average ambient temperature frequently exceeds 25°C but also for temperate countries exposed to summer heat waves. For example, HS leads to a global yearly economic loss of about $300 million in the US pig industry [1]. According to the predicted consequence of global warming, especially the increase in the frequency and the severity of summer heat waves, it is clear that HS is not only a current but also an emerging issue for the world pig production. Under these circumstances, a better understanding of the impact of HS on swine physiology and metabolism and the promotion of innovative strategies are needed to limit the economic consequences of HS on pig farms profitability.

Several studies showed that the pig’s thermoregulatory responses for avoiding HS are activated above 25°C [2]. This threshold temperature varies according to various factors including animal related factors (genotype, body weight, physiological stage) and environmental factors like relative humidity. Regarding the genotype, genetic selection for high lean deposition rate in growing pigs has resulted in an increased sensitivity to high ambient temperatures [3]. In HS conditions, a significant reduction of the voluntary feed intake is generally observed in swine. This response is considered as the main adaptation mechanism for reducing metabolic heat production [4], which has negative subsequent effects on growth performance [5].

Various mitigation options are applied against HS, including costly heat abatement (spray or floor cooling) and/or nutritional strategies. In practice, animal responses to HS are highly variable within a population and a part of this variability has a genetic basis [6,7]. Given this definition, robust pigs are animals that combine high production levels and adaptation capability in a wide variety of environmental conditions [8]. Identifying novel diagnostic tools or biomarkers of sensitivity to HS will be an important step towards the management of HS in pigs. These new tools could be used to prevent HS related problems, to improve the efficiency of current coping options by targeting the most thermosensitive pigs and/or to improve genetic program by the identification of genomic variants associated with heat tolerance. In the objective of diagnostic and development of biomarker tools based on metabolomic analyses, plasma is a suitable target tissue as it is easy to obtain with minimal stress for animals. Plasma is also the main carrier of different metabolites whose concentrations can be affected by heat stress [9]. Among the existing technics, the ^1H^NMR method has the ability to accurately quantify metabolites from a complex biological matrix (blood plasma) with high precision and with a fast and cheap sample preparation procedure [10]. The objectives of the present study are: 1/ to propose a strategy to evaluate HS sensitivity in ten pig sire families (SF) using performance of descendants raised either in temperate (TEMP) or in tropical (TROP) environments, and 2/ to build a predictive model of pig sensitivity to HS based on plasma metabolomic signature, using the sparse partial least square discriminant analysis (sPLS-DA) statistical approach.

## Materials and methods

All experimental procedures involving the use of animals were approved by the local Animal Care and Use Committees at the Poitou-Charentes and French West Indies and Guyana INRA centers (authorizations N° CE202-9 and 69-2012-2, respectively).

### Experimental design

Data used in the present study were obtained in a population designed to examine the genetic background of heat tolerance in growing pigs [11].To sum up, genetically related Large White sows reared in two different locations (temperate and tropical areas) were sired with the same ten crossbred Large White×Créole boars. A total of 634 backcross pigs from 60 Large White sows (raised in 11 contemporary groups or batches) and 664 BC from 70 Large White sows (12 batches) were obtained in temperate (TEMP, INRA experimental facility Le Magneraud, GenESI, Surgères, Charentes, France) and tropical (TROP, INRA experimental facility PTEA, Petit-Bourg, Guadeloupe, France) conditions, respectively. As it was extensively described and discussed in [12], the surrounding temperature and humidity are higher in TROP region than in TEMP over the years. Several studies showed that the combination of both factors increases the actual temperature perceived by livestock especially in tropical humid conditions [3,13,14]. In this study, the effective ambient temperature was quantified by calculation of a thermal humidity index (THI) which combines the average daily ambient temperature and the average daily relative humidity [12]. Based on the THI calculations, pigs raised in tropical conditions were exposed to heat stress during the experiment (S1 Fig). Finally, on average, each crossbred boar produced of average 56±5 offspring per environment, females and castrated males. Pigs were weaned at 4 weeks of age (week 4) (26.9 ± 1.7 d). During the growing period (between weeks 10 to 23), pigs of a given batch were housed in 6 pens of 10 animals. The animal phenotyping period started after one week of adaptation (week 11) and ended at week 23. Each pen was equipped with nipple drinkers and animals had free access to water. They were fed ad libitum with a commercial diet presented as pellets and formulated to meet or exceed the nutritional requirements of growing-finishing pigs according to the standard INRA recommendations.

### Data recording

#### Phenotypic measurements

All pigs were weighed (BW) from week 11 (BW11) to week 23 (BW23). The average daily BW gain (ADG, g/d) was calculated by dividing the BW gain from 11 to 23 weeks of age divided by the period duration in days. On week 23, the backfat thickness was measured ultrasonically (Agroscan, E.C.M, Angoulême, France) at 6 different sites, measured directly above the point of the elbow, last rib (P2 site) and last lumbar vertebra locations, respectively, and taken 5 cm off the midline on each side of the pig. The average back fat thickness (ABFT, mm) was the average value of these 6 measurements. Individual daily feed intake was recorded using electronic feeders (ACEMA 128, ACEMO, Pontivy, France) either only during test weeks 11–12, 15–16 and 19–20 (defined as period 1) for half of the pigs or only during test weeks 13–14, 17–18 and 21–23 (defined as period 2) for the other pigs. During the remaining test weeks, pigs had free access to a conventional feed dispenser. We calculated the average daily feed intake (ADFI) over the whole trial based on these measurements, as proposed by [15]. Rectal temperature (RT) and skin temperature (ST) were measured at 23 weeks of age on resting animals. RT was measured using a digital thermometer (Microlife Corp., Paris, France), and ST was measured on the back at the P2 site using a skin surface thermocouple probe (type K, model 88002K-IEC, Omega Engineering Inc., Stamford, ST, USA) connected to a microprocessor-based handheld thermometer (model HH-21, Omega Engineering Inc).

#### Plasma sample preparation

From the 1298 pigs, data from 560 animals sampled for plasma at 23 weeks of age in TEMP climate were used in the present study. For each sampling, the same protocol was applied. Pigs were restrained in a V shaped system for avoiding stress. Plasma samples were obtained by a jugular vein puncture using a 9 ml-vacutainer plasma collection system that contains heparin. This vein puncture procedure required less than 2 min per pig. Plasma samples were centrifuged at 2,500 g for 20 min to separate plasma. Plasma samples were stored frozen (-80°C) until they were assayed.

#### ^1H^Nuclear Magnetic Resonance (^1H^NMR) spectroscopy analysis

Generation and normalization of metabolomics spectra were carried out on MetaToul platform (Toulouse metabolomics and fluxomics facilities, www.metatoul.fr). Plasma samples (200 μL) were diluted with 500 μL of D_2_O and centrifuged at 5,000 g for 10 min at 4°C before they were placed in 5 mm ^1H^NMR tubes. All ^1H^NMR spectra were obtained on a Bruker DRX-600-Avance ^1H^NMR spectrometer operating at 600.13 MHz for ^1H^ resonance frequency using an inverse detection 5 mm ^1^*H*-^13^*C*-^15^*N* cryoprobe attached to a CryoPlatform (the preamplifier cooling unit). The ^1H^NMR spectra were acquired at 300 K using the Carr-Purcell-Meiboom-Gill (CPMG) spin-echo pulse sequence with pre-saturation, with a total spin echo delay (2nτ) of 240 ms to attenuate broad signals from proteins and lipoproteins. A total of 128 transients were collected in 32,000 data points using a spectral width, a relaxation delay and an acquisition time of 20 ppm, 2.5 sec, and 2.28 sec, respectively. The spectra were Fourier transformed by multiplication of the free induction decay FIDs by an exponential weighting function corresponding to a line-broadening of 0.3 Hz. All spectra were manually phased and baseline corrected, and referenced to 3-trimethylsilylpropionate TMSP using the Bruker TopSpin 2.1 software (Bruker, GMBH, and Karlsruhe, Germany). Data were reduced using the AMIX software (version 3.9, Bruker, Rheinstetten, Germany) to integrate 0.01 ppm wide regions (i.e., buckets) corresponding to the δ 9.00–0.70 ppm region for plasma samples. The 5.10–4.50 region, comprising water resonances, was excluded. To account for differences in the amount of used material, each integrated region was normalized to the total spectral area [16]. Spectral assignment was based on matching data to reference spectra in a home-made reference database, as well as with others database (http://www.bmrb.wisc.edu and http://www.hmdb.ca), and reports available in the literature [17,18]. In practice, each identified metabolite can correspond to several buckets. Finally, each plasma metabolomic profile was described with 333 and 112 annotated and non-annotated buckets, respectively. Non-annotated buckets were labeled “unknown”.

### Statistical analyses

#### General purpose of statistical analyses

Statistical analyses were performed with the R software [19]. First of all, the phenotypic and metabolomic data were adjusted for undesirable batch and sex effects. All the subsequent statistical analyses were made on the surrogate adjusted variables. Analysis of variance was done with the Type III ANOVA. The metabolomic data and metadata that were used in this study are provided in the S1 Data File.

**Fig 1:**
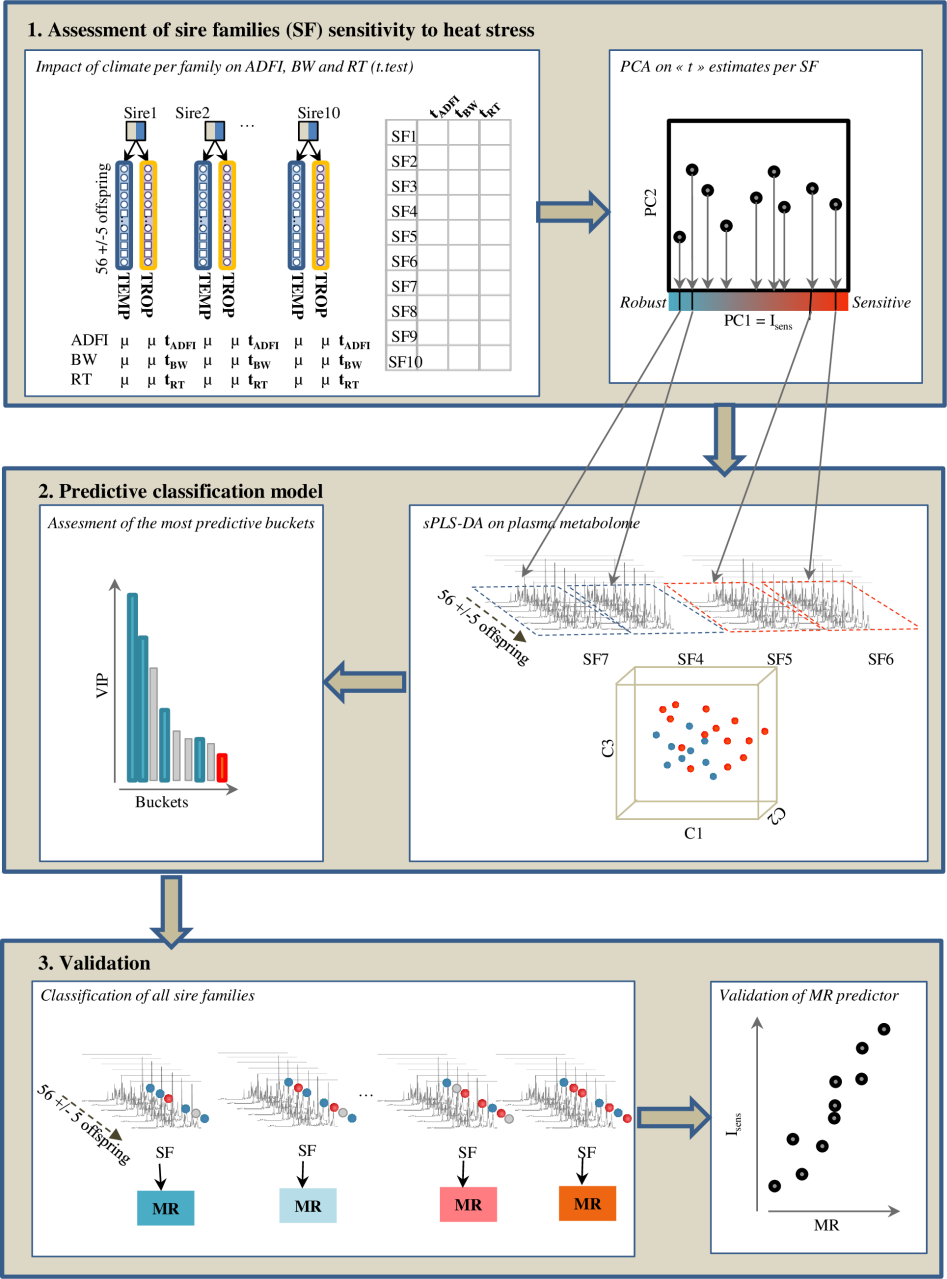
Statistical workflow. Statistical approach that lead to: 1) assess sensitivity of each SF to TROP climate according to a composite index of sensitivity (I_*sens*_) that took into account the between-climate variations of average daily feed intake (ADFI), body weight (BW) at 23 weeks and rectal temperature (RT) at 23 weeks, 2) Build a supervised classification model on metabolomic data between the robust and climate sensitive groups by *s*PLS-DA, 3) predict the membership rate (MR) of the other SF to the sensitive group using *s*PLS-DA. The relevance of MR interpretation as predictive index of sensitivity was confirmed through its highly significant correlation with index of sensitivity (I_*sens*_).

The present study aimed to assess and predict the sensitivity of sire families to HS encountered in TROP region using metabolomic records obtained in TEMP conditions. As shown in Fig 1, three main steps were taken:

1. We firstly quantified the actual average sensitivity of SF to TROP climate using phenotypic traits.
2. In a second step, we selected the 4 SF that showed extreme degrees of sensitivity (2 SF) and robustness (2 SF) to HS to build a predictive classification model using the profiled metabolomes in TEMP climate and sPLS-DA method. The proportion of offspring predicted as contributing to the sensitivity of the SF was considered as a predicted index of sensitivity of that SF.
3. Ultimately, we validated the ability of our classification model to predict the sensitivity of SF by testing the correlation between the observed and predicted average sensitivity of the 6 remaining SF.

#### Phenotypic and metabolomic data adjustment

In order to adjust the metabolomic and phenotypic data sets for batch and sex effects, we used the empirical Bayesian method described in [20] accounting for climate, sire family (SF), batch and sex effects. To validate the adjustment of the data, we used the principal variance component analysis (PVCA) method to determine the proportion of variance of each effect before and after adjustment [21]. Before adjustment, the percentage of variance due to climate, SF, batch and sex effects on phenotypic data were 18.9, 4.4, 5.3 and 7.6%, respectively (S2A Fig). For metabolomic data, the corresponding values were 4.9, 4.4, 6.5 and 0.8%, respectively (S2C Fig). After adjustment, S2B and S2D Fig showed that the contributions of batch and sex effects were successfully removed from the variance of data sets.

#### Assessment of sensitivity to heat stress

As shown in the [11], ADFI, BW and RT are particularly affected by climate. Additionally, it’s commonly admitted that the reduction of feed intake in case of heat stress is a strategy of thermal adaption to reduce internal temperature. First, to quantify the degree of the sensitivity of each SF to the TROP climate (n = 46 to 65 observations per SF in TEMP and 41 to 60 observations per SF in TROP), the familial between-climate deviations were assessed for ADFI, BW and RT using *t-*tests (with Welsh correction) (Fig 1). Unlike the Student’s *t-*test, the Welch’s *t-*test is designed to compare two populations with unequal variances. Changes of average growing performances and ability to maintain internal body temperature between TEMP and TROP environments within each SF were thus considered as indicative of the familial sensitivity. From these *t*-tests, a 10 sires x 3 traits matrix of the *t* statistics values (S1 Table) was built. For convenience, the “*t”* statistics were written *t*_ADFI_, *t*_BW_ and *t*_TR_ for ADFI, BW and RT, respectively. Thus, each SF was characterized by three unit-free variables. We performed a principal component analysis (PCA) on this matrix to assess the components of SF differences in HS sensitivity by considering performance (*t*_ADFI_ and *t*_BW_) and thermoregulation (*t*_RT_) related traits. We retained the first component of the PCA (PC) as indicator of sensitivity to HS, the scores of each SF for the first PC being considered as a composite index of familial sensitivity (I_*sens*_).

#### Predictive classification model

In order to predict sensitivity to TROP climate, our strategy was to use ^1H^RMN metabolomic data obtained in TEMP climate to predict the SF sensitivity status to TROP climate. The most extreme SF regarding sensitivity were identified based on I_*sens*_ values. The metabolomics data of the pigs from these SF in TEMP climate were used to train a classifier that identified the most predictive buckets of sensitivity. The model was validated using cross validation (CV). Details about the different steps of this analysis are described below.

According to the index of sensitivity, we built two extreme groups: 1) a robust group (n = 102 pigs) composed of the 2 most robust SF according to their I_*sens*_ and, 2) a sensitive group composed of the 2 most sensitive SF according to their I_*sens*_ (n = 121 pigs)_._ The metabolomic data of robust and sensitive groups in TEMP climate were used as a calibration set in a multivariate discriminant analysis performed via *s*PLS-DA, discriminating robust/sensitive families. Contrary to a classical PLS-DA, *s*PLS-DA, as described and implemented in the R package mixOmics, performs variable selection on the basis of a soft thresholding that approximates the Lasso penalization [22]. The purpose of this variable selection is to remove the less informative buckets and to make the interpretation of the model easier. To choose the optimal number of buckets to select, we used the method described by Lê Cao et al (2011) [23]. We set up the tuning process until obtaining the minimum number of buckets between 5 and 20 that allowed the lowest balanced error rate (BER). At the default decision threshold of sPLS-DA (0.5), BER is an average error rate of prediction. BER is a suitable metric of a model's inaccuracy in case of imbalanced groups.

BER=1−0.5×(modelsensitivity+modelspecificity)

The sensitivity of the model (true positive rate) is the number of animals truly predicted as sensitive to HS over the actual number of animals sensitive to HS. The specificity (true negative rate) of the model is the number of animals truly predicted as robust to HS over the actual number of animals robust to HS. Finally, the sensitivity and 100%-specificity (false positive rate) were computed at different decision thresholds and were plotted as the receiver operating characteristic curve (ROC). The area under the curve (AUC) showed the average prediction performances overall the varying decision thresholds. The closer the AUC is to 1 the better the accuracy of the model is [24].

Once the optimal number of buckets was chosen, the sPLS-DA model was re-learnt with it and provided loading values for every selected bucket. A large absolute loading value of a bucket reflects a high covariance between that bucket and the variable to explain. Moreover, the variable influence in projection (VIP) was computed for each component of sPLS-DA. Variables that show a VIP > 1 are usually considered as significant contributors to the prediction model [25].

Additionally, we provided a stability criterion of selected variables in the model. Variable selection was repeated across a 10-fold CV and different sets of selected buckets were obtained at each replicate of CV. The reproducibility of the variable selection across the 10-fold CV was assessed by means of its frequency of selection, similarly as what was proposed for bootstrap method by [26,27]. The stability measure ranges between 0 and 1, and is equal to one for the buckets that are always selected. Conversely, buckets can be selected in one run over the 10 and the stability measure is thus equal to 0.1, meaning that the selection of this bucket is weakly reproducible.

#### Validation of the predictive model

Metabolomic profiles from descendants of the 6 other SF (n = 337 pigs) were used as a validation set to assess the predictive ability of the model. The validation method was declined in two steps. First, prediction of classes (robust or sensitive) was computed for each pig. A membership rate (MR) to the sensitive group was then calculated for each SF in the validation set. This MR was equal to the percentage of animals predicted as sensitive in the SF, and was interpreted as a predicted indicator of sensitivity to HS for the SF. In a second step, a Pearson correlation test was performed between the MR (predicted sensitivity) and the I_*sens*_ (real sensitivity).

From the predictive model, a number of buckets and metabolites with VIP > 1, i.e. contributing most to the discrimination between sensitive and robust families to HS based on the TEMP samplings, were selected. We assessed the correlations between the median values per SF of these selected buckets in TEMP climate and the average phenotypic traits per SF both in TEMP and TROP climates.

## Results

### Impact of climate on animal performance and thermoregulatory responses

All performance and thermoregulation traits were significantly affected by climate. For most of the traits, the effect of climate differed according to the SF (P < 0.01, S2 Table) suggesting high genetic × climatic environment interactions. The final BW and ADFI decreased significantly in TROP climate in comparison to TEMP climate (S2 Table). Conversely, RT increased significantly in TROP compared to TEMP climate (P < 0.001, S2 Table). The increase of RT in TROP environment was dependent on the SF (P = 0.03, S2 Table).

### Assessment of sensitivity to heat stress

**Fig 2:**
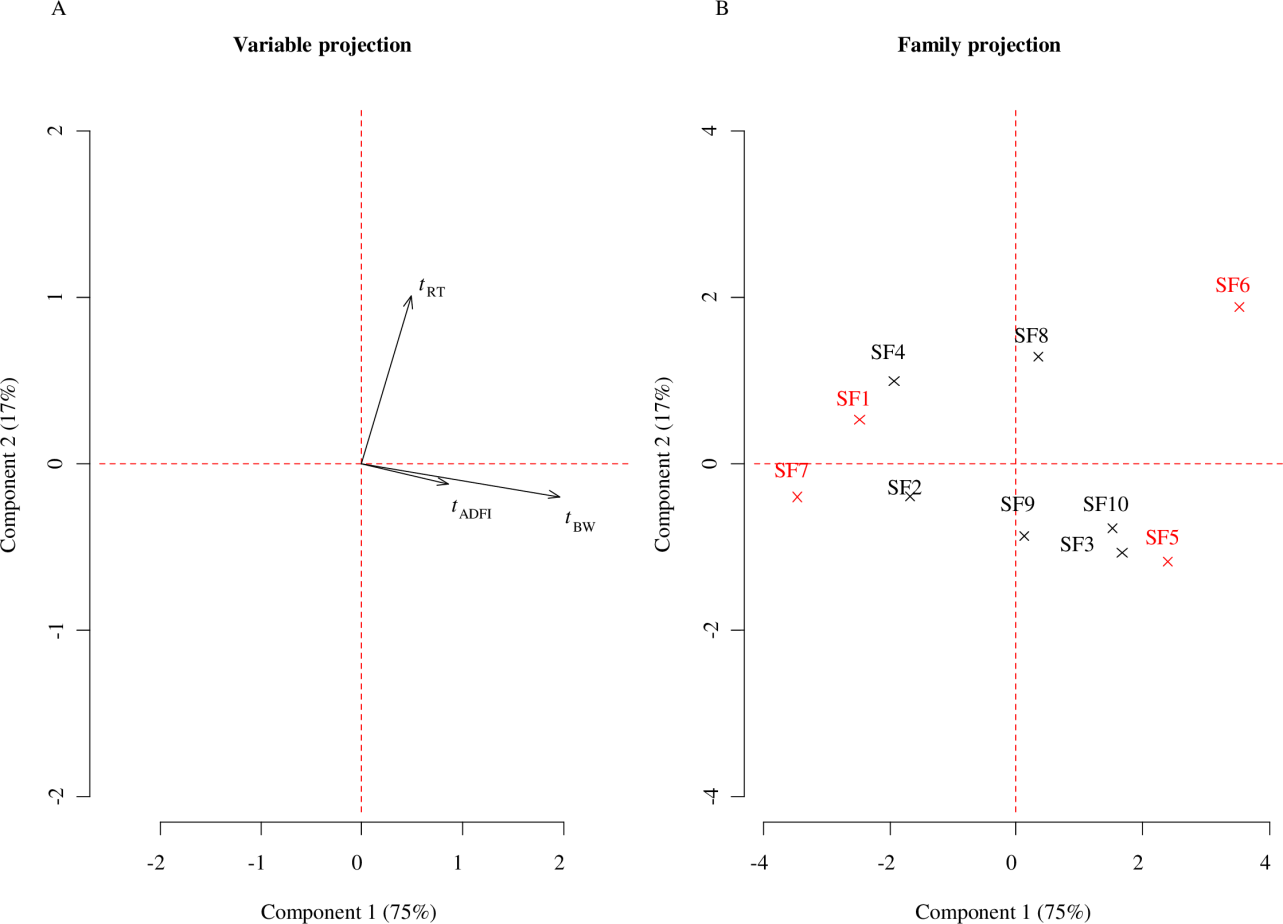
Multivariate description of the sensitivity to heat stress at the sire family (SF) level. PCA was performed on *t* statistics between TEMP and TROP climates for each SF (*t*_BW_, *t*_ADFI_, *t*_RT_ for live body weight, average daily feed intake and rectal temperature, respectively). (A) and (B) represent the variables and samples PCA projections on the 2 first component.

For each Sire Family (SF), the variation between climates of each quantitative phenotype was assessed with a Welch’s *t*-statistic, producing a matrix of *t* values (S1 Table). The first and second components of the PCA applied to this matrix explained 75% and 17% of the total inertia, respectively. The contributions of *t*_BW_, *t*_ADFI_, and *t*_RT_ to the first component were 75, 17, and 8% respectively (Fig 2), with increased values on the first components associated to increased *t* (Fig 2A). The index of sensitivity (I_*sens*_) to the environment of each SF, quantified as its coordinate on the first component. Thus, I_sens_ increased from negative to positive values. Families SF6 and SF5 (I_*sens*_ = 3.5 and 2.4, respectively) were found to be the most sensitive and were selected to constitute the sensitive reference group (Fig 2B). On the opposite, SF7 and SF1 (I_*sens*_ = -3.4 and -2.5, respectively) were found the most robust and constituted the robust reference group.

The comparison of the average performances of progeny between the robust and the sensitive groups within the TEMP climate did not show significant difference for BW, ADG, ADFI (P > 0.05) (Table 1). However, significant group differences were observed for the three traits in TROP, with higher ADFI and BW in the robust group (Table 1). Similarly, ADG was equivalent in TEMP for both groups, and larger in the robust SF progeny than in the sensitive ones (P<0.001). However, ST did not differ between groups in each climate, FCR tended to be greater in the sensitive group than in robust group in TEMP climate (P < 0.01), but not in TROP conditions. Finally, ABFT was significantly higher in the sensitive group only in TEMP climate.

**Table 1:** Phenotypic means of robust and sensitive groups in TEMP and TROP climates.

Climate	TEMP		TROP			
**Group**	**Robust (n = 102)**	**Sensitive (n = 121)**	**Robust (n = 115)**	**Sensitive (n = 83)**	**RMSE**	**Statistical significance**^**2**^
**Traits used to compute sensitivity**^**1**^						
**ADFI (kg/d)**	2.21^a^	2.29^a^	1.85^b^	1.64^c^	0.42	C***, SF*, C x G*
**BW (kg)**	102^a^	101^a^	88^b^	79^c^	9	C***, SF***, C x G***
**RT (°C)**	39.3^a^	39.2^a^	39.4^b^	39.5^b^	0.3	C***, C x G^#^_,_ C x SF(G)^#^
**Other traits of interest**^**1**^						
**ADG (g/d)**	814^a^	799^a^	779^b^	695^c^	136	C***, SF***, C x G***
**ST (°C)**	34.4^a^	34.3^a^	35.7^b^	35.5^b^	0.7	C***, SF***
**FCR (kg/kg)**	2.73^a^	2.90^a^	2.42^b^	2.39^b^	0.54	C***, SF*, C x G ^#^
**ABFT (mm)**	19.2^a^	21.6^b^	16.0^c^	15.5^c^	2.7	C***, SF***, C x G ***, C x SF (G)^#^

^1^ ADFI = Average daily feed intake, BW = Body weight, RT = Rectal temperature, ADG = Average daily weight gain, FCR = Feed conversion ratio, ABFT = Average back fat thickness, ST = Skin temperature.

^2^ Data were analyzed with an ANOVA (type III) including the effect of climate (C), groups (G), sex (S), and sire family (SF) and interactions cofactors. Only trend or significant results are mentioned with ***: P < 0.001, *: P <0.05 and #: P < 0.10.

Different letters indicate statistical significance (P < 0.05) in Tukey post hoc test.

The crosses (x) indicate interaction between factors.

C x SF (G) indicates interaction between climate and SF nested in G groups.

**Fig 3:**
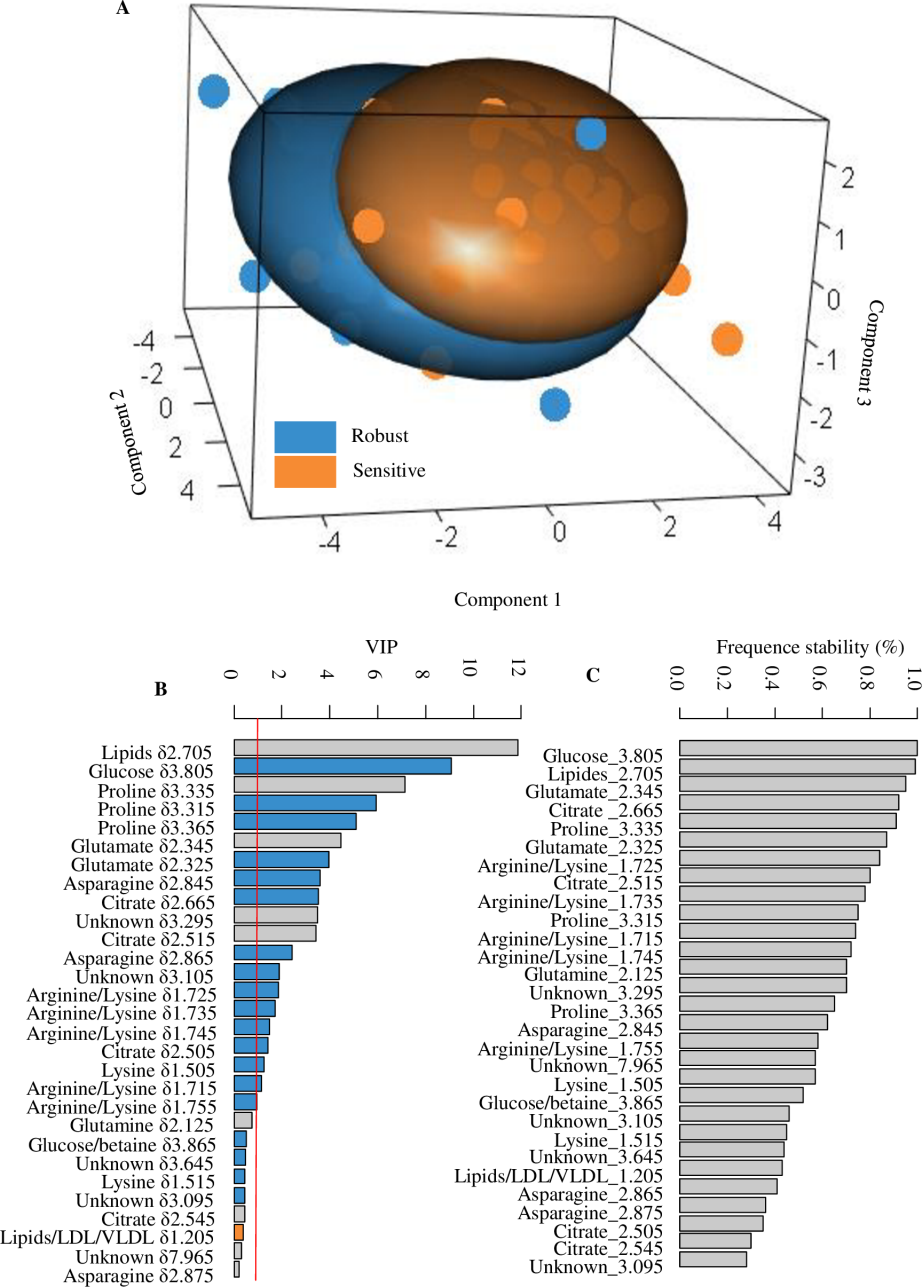
sPLS-DA between robust vs sensitive groups. (A) Projection of animals on the 3 components of the *s*PLS-DA on metabolomic data between robust (blue) and sensitive groups (orange). Spheres are a 3D representation of confidence ellipses (level 95%). (B) VIP (variable importance projection) plot that shows the variable importance in the *s*PLS-DA model over the 3 components. Variables with a VIP > 1 (red line) were considered as highly important predictors in *s*PLS-DA. The blue and orange bars are the buckets that showed a significant higher median value (Wilcoxon test: FDR < 0.05) in the robust or in the sensitive group, respectively. The grey bars indicate buckets that are not significantly different between the robust and the sensitive group (Wilcoxon test: FDR > 0.05). (C) Frequencies stability of selection of variable across the 100 models of CV.

A *s*PLS-DA was used in order to discriminate the extreme sensitive and robust groups using metabolomic data obtained in TEMP. The tuning step of the number of components to select showed that 3 components were necessary to lower the balanced error rate (BER) (S3 Fig). The visual analysis of the graphical outputs of the tuning step showed that the BER for the first component remained stable whatever the number of selected buckets (S3A Fig). The second component showed that the lowest BER was obtained with 5 buckets only. Finally, the lowest BER was obtained with 19 buckets for the third component (S3A Fig). Thus, the *s*PLS-DA model was built with a selection of 5, 5 and 19 buckets on the 1^st^, 2^nd^ and 3^rd^ components, respectively. The 1^st^, 2^nd^ and 3^rd^ components of the sPLS-DA explained 0.17, 0.07 and 0.06% of the metabolome variance, respectively (Fig 3A). The cross validation showed a BER of 0.22. The area under the ROC curve was estimated to 0.83 (P < 0.001) (S3B Fig). The most important variables in the model (i.e., variables with a VIP > 1) were related to some amino acids such as glutamate (*δ*2.345, *δ*2.325), proline (*δ*3.335, *δ*3.315, and *δ*3.365), lysine (*δ*1.505), lysine/arginine (*δ*1.725, *δ*1.735, *δ*1.745, *δ*1.715, and *δ*1.755), and asparagine (*δ*2.845, *δ*2.865) (Fig 3B). Among the selected predictors, we also found the buckets *δ*2.705 (VIP > 10) and *δ*1.205 (VIP < 1) that were related to lipids and Lipids/LDL/VLDL, respectively. The set of important predictors also included glucose (*δ*3.805), and citrate (*δ*2.665, *δ*2.515, *δ*2.505). Variable frequence stability assessment showed that buckets related to glucose (*δ*3.805), lipids (*δ*2.705), glutamate (*δ*2.345), citrate (*δ*2.665, *δ*2.515), proline (*δ*3.335), glutamate (*δ*2.325), and arginine/lysine (*δ*1.725) were highly stable (stability measure > 0.8) (Fig 3C).

In addition, we performed a Wilcoxon rank test and the P values were adjusted for FDR (False Discovery Rate) to assess significance of the differential abundance of each bucket between sensitive and robust groups. The results showed significantly higher levels in the robust group compared to the sensitive group for the buckets related to glucose (*δ*3.805), proline (*δ*3.315 *δ*3.365), glutamate (*δ*2.325), asparagine (*δ*2.845, *δ*2.865), citrate (*δ*2.665, *δ*2.505), lysine/arginine (*δ*1.725, *δ*1.735, *δ*1.745, *δ*1.715, *δ*1.755), lysine (*δ*1.505), and an unknown bucket (*δ*3.105) (FDR < 0.05, VIP > 1) (Fig 3B). The bucket related to LDL/VLDL (*δ*1.205) was found significantly more abundant in the sensitive group than in the robust group (Fig 3B).

**Fig 4:**
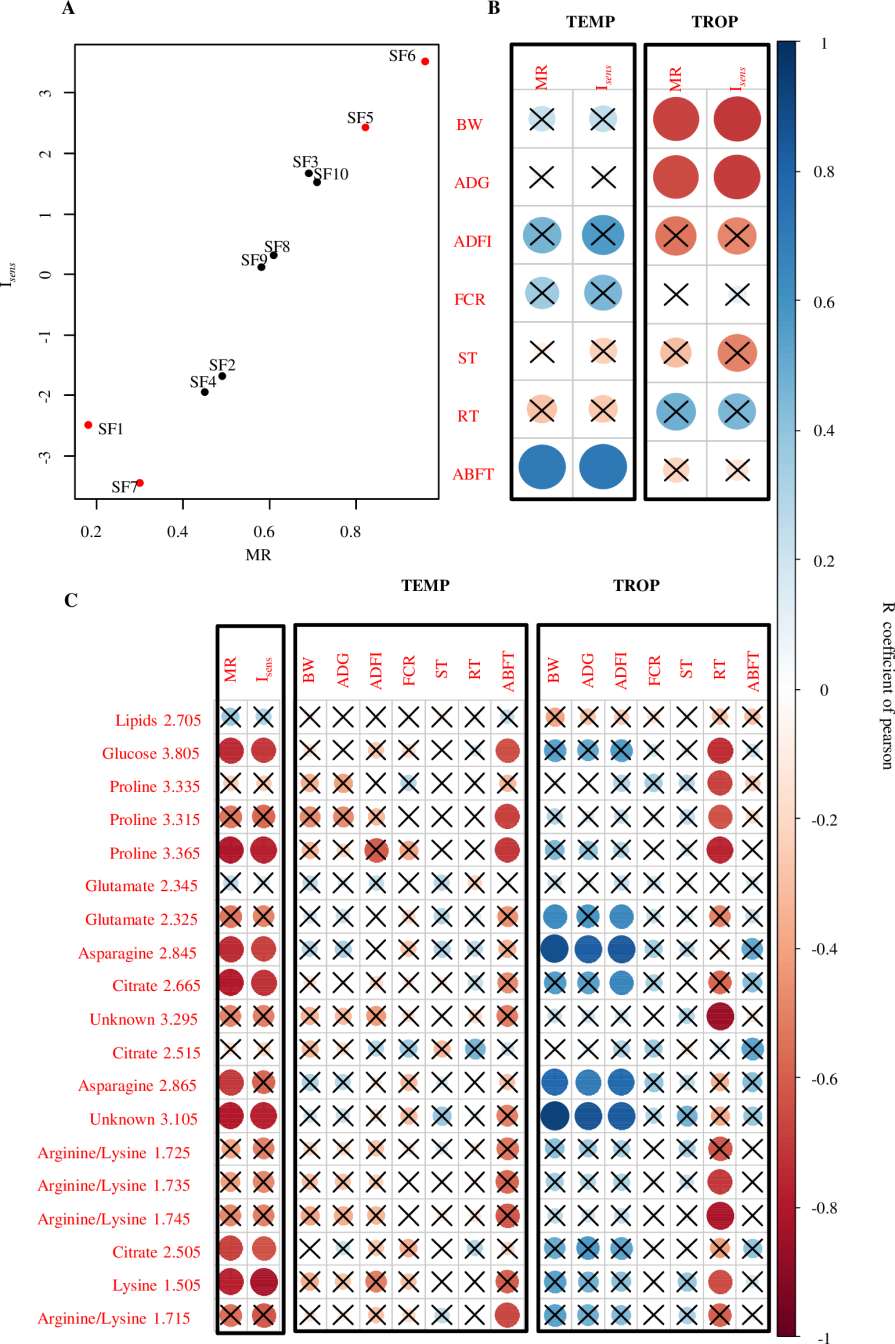
Correlations analysis. (A) The plot shows correlations between the MR of SF to sensitive group and the I_*sens*_ (r = 0.96, P < 0.0001 for Pearson correlation test). (B) The plot shows correlations of MR and I_*sens*_ against the mean feed conversion rate (FCR), average daily weight gain (ADG), weight, rectal temperature (RT), skin temperature (ST), back fat thickness (ABFT) per SF both in TEMP and TEMP climate. Blue and red circles showed positive or negative correlation respectively. (C) The plot shows correlations between the median values selected buckets with a VIP > 1 per SF in TEMP climate and mean values per SF of BW, ADG, ADFI, FCR, ST, RT, ABFT, MR, and I_*sens*_ either in TEMP or in TROP climate. The size of circle is proportional to the absolute value of R coefficient of correlation of Pearson. The significance of correlation is P < 0.05 (Pearson correlation test) and the crosses indicate non-significant P values (P > 0.05).

Metabolomes of all animals from training and validation sets were submitted to sPLS-DA model to predict their class membership (robust or sensitive). Among the already identified robust group, the membership rate (% of sensitive pigs) was 30 and 18% of the animals from SF7 and SF1, respectively. Conversely, 82 and 96% of the animals from SF5 and SF6 were predicted as sensitive, respectively. For the remaining validation set of SF, 49 and 45% of the animals from SF2 and SF4 were predicted as sensitive, respectively, and 58, 61, 64 and 71% of the total number of the animals from SF9, SF8, SF3 and SF10, respectively. Finally, we observed a strong significant positive correlation between the I_*sens*_ index and MR of tested families (r = 0.97, P < 0.01) (Fig 4A). Correlation coefficients were calculated between MR, I_*sens*_ and traits related to pig performance in both climates. In the TEMP climate, MR and I_*sens*_ were positively correlated to the mean SF value of ABFT (r = 0.71 and 0.71, respectively, P < 0.05) (Fig 4B). In the TROP climate, MR and I_*sens*_ showed significant negative correlations with BW and ADG (MR: r = -0.67 and -0.65 respectively; I_*sens*_: r = -0.71 and -0.69, respectively; P < 0.05) (Fig 4B). All other correlations were not significantly different from zero.

We observed a significant negative correlation between MR or I_*sens*_ with the median values of selected buckets (VIP > 1) for buckets related to glucose (*δ*3.805), proline (*δ*3.365), asparagine (*δ*2.845, *δ*2.865), lysine (*δ*1.505), citrate (*δ*2.665, *δ*2.505), and one unknown (*δ*3.105) bucket (P < 0.05) (Fig 4C). The correlation analysis between these median values of selected buckets and the familial mean of phenotypes showed that buckets related to glucose (*δ*3.805), proline (*δ*3.315, *δ*3.365) and arginine/lysine (*δ*1.715) were negatively correlated with average familial ABFT in the TEMP climate (P < 0.05) (Fig 4C). Additionally, the median values of buckets related to proline (*δ*3.335, *δ*3.315, *δ*3.365), glucose (*δ*3.805), arginine/lysine (*δ*1.745, *δ*1.735), and one unknown (*δ*3.295) showed a significant negative correlation with the average value of RT in the TROP climate (Fig 4C). Finally, the median values of buckets related to asparagine (*δ*2.845, *δ*2.865) and one unknown bucket in TEMP showed a significant positive correlation with familial ADG, BW and ADFI in the TROP climate (Fig 4C). In TEMP, BW, ADFI, FCR, ST and RT showed no correlation with the buckets abundancies, neither FCR, ST and ABFT in TROP.

## Discussion

Preventing the adverse effects of HS on animal productivity will be an increasingly challenging goal for many livestock production systems in the future. Data obtained in the present study confirm the negative impact of hot and humid tropical environment on growing-finishing pig performances, with a significant reduction of voluntary feed intake leading to subsequent negative effects on growth [28]. In the present study, ADG measured between 11 and 23 weeks of age was reduced by about 10% in TROP compared to TEMP (759 vs. 836 g/d). This result is in agreement with previous results published by Gilbert et al (2012) (-11%; 680 vs. 780 g/d) [29]. Based on the average performance of sire progeny, the ranking of the sires changed according to the climatic environment. In other words, this suggests that the best sires in TEMP environment would not be the best ones in TROP conditions. Such re-ranking of performance between thermoneutral and warm conditions has already been observed in cattle by Bradford and al (2016) [30]. Our results confirm the existence of sire effect by climatic environment interactions for most of the performance traits. In practice, genotype × environment interaction (G × E) is commonly considered as a potential source of inefficiency in pig breeding programs. As suggested by Knap and Su (2008), G × E could be better exploited for promoting innovating breeding programs [31]. Nowadays breeding programs are generally transnational and consequently, the goals of such programs are to breed animals that can perform well in a variety of environments. The improvement of animal robustness can be achieved either by a direct inclusion of robustness indicators in a breeding goal or by selecting animals for increased levels of production in diverse environments, i.e., lower environmental sensitivity. In the case of HS sensitivity, these options require an access to easily measurable heat adaptation related-traits or to performance traits in various climatic environmental conditions. In practice, the estimation of the genetic values of a breeding animal in a large scale of climatic environments can be costly. Additionally, the estimation of the genetic merit obtained can be specific to the environments of the testing protocol. Traits to describe heat tolerance relate the ability of the animals to fight against HS and are often based on body temperature measurements (rectal and skin temperatures) or on other physiological parameters such as heart or respiratory rates. These traits display genetic variation within and between breeds, demonstrating the possibility for breeding animals in order to obtain heat tolerance responses [32]. However, according to the genetic antagonism between heat adaptation and performance related traits, selection for heat tolerance may have unfavorable consequences on growth performance [33]. Moreover, heat adaptation traits are generally difficult and costly to measure. One alternative option could be to develop a diagnostic tool for sorting the future reproducers in the nucleus herd better suited for the specific needs of commercial herds localized in normal or hot climatic conditions. The main objective of this study was to provide possible biomarkers for a future diagnostic tool based on the fitness evaluation of sires in two contrasted climatic environments (TEMP vs. TROP) using plasma ^1H^RMN metabolic information obtained from blood samples collected in TEMP. For this purpose, we assessed the reliability of plasma metabolome signature for prediction of a sensitivity index to heat tolerance in swine.

In pig breeding, robustness was defined by Knap et al (2005) as “the ability to combine a high production potential with resilience to stressors, allowing for unproblematic expression of a high production potential in a wide variety of environmental conditions” [8]. This definition can be applied for heat tolerance as the ability of pigs to maintain production and adaptation in a wide variety of climatic environmental conditions[34,35]. In the present study, the sensitivity was assessed with a composite index computed from the between-climate deviations of consumption, body weight and rectal temperature. A similar approach has been already used in the context of socioeconomic study on human well-being measurement in the Netherlands [36]. However, although this generic *t*-test-PCA based approach can be applied to a wide variety of scientific fields to establish a multivariate measurement of communities’ responsiveness to a specific factor (here sire origin and climatic condition), such composite indices are not generalizable reference values as they depend on the chosen initial variable, on the methods of measurement and on the sample size. Thus, our composite index, called “index of sensitivity” (or I_*sens*_), should be interpreted as a relative measurement of sensitivity between SF involved in our experimental set and accordingly to our definition of sensitivity.

The main objective of our study was to propose an effective predictive tool to assess the environmental sensitivity (i.e., I_*sens*_) with a combination of blood metabolites obtained from a blood sample collected in thermoneutral conditions. Huiqing et al (2005) demonstrated that using extreme patient samples in the training phase of statistical modeling leads to improved prediction performances of survival time and risk of tumor metastasis or recurrence in comparison with models that are trained with non-extreme samples [37]. A similar approach was used in our study and SF with extreme I_*sens*_ indexes were taken as reference groups to model robustness and sensitivity, being thus the most informative for selecting relevant features in metabolomic data for predicting heat tolerance.

Our results highlighted differences in the average performance between the extremely sensitive and the robust groups. The sensitive group was found to have a lower feed efficiency (higher feed conversion ratio) and to be fatter at the end of the control period in TEMP. This high fat deposition could be considered as a direct consequence of the lower feed efficiency. However, average growth rate and final BW were similar in both groups. Our results suggest a possible link between feed efficiency and the ability to cope with thermal stress in pigs. Similarly, Hermesh et al (2015) suggested that genetic selection on feed efficiency may have an impact on robustness of pigs in stressful conditions [38]. From a comparison between divergent lines selected for a low or a high feed efficiency using the residual feed intake as a selection criterion, it has been demonstrated that the line with a high feed efficiency had a lower basal metabolite rate [39]. Based on the hypothesis that this reduced heat production would confer a better heat tolerance to the high efficient line, the thermoregulatory responses of both lines were measured in hot conditions [40]. From these studies, there is still no clear evidence of the association between variability in feed efficiency and the sensitivity to HS but the initial hypothesis of adverse correlations between production and response to HS, mentioned in [29], might not be sustained.

Among the numerous supervised learning techniques, the partial least squares-discriminant analysis (PLS-DA) is one of most widely used chemometric multivariate method in ^1H^NMR data analysis. PLS-DA performs well in case of a large number of explanatory variables with the presence of collinearity between variables [41]. To limit the number of predictors, to improve prediction accuracy and to avoid over-fitting, sparse PLS-DA performs a variable selection in a Lasso-like manner [42]. The overall prediction error rate of 22% could be due to the quality of the model and to the limits of blood metabolome to predict robustness more accurately. However, this reasonable prediction error rate may be partly imputed to the presence of potentially sensitive individuals within robust SF of the training set. Indeed, the I_*sens*_ index is a continuous variable indicating that sensitivity is not an “all or nothing” phenotypic trait but that it spreads along a wide range of values. The observed strong significant correlation between the I_*sens*_ index and the proportion of animals predicted as sensitive (i.e., Membership Rate or MR) within each of the 10 SF confirmed the reliability of the selected metabolomic markers by our model for prediction of the average sensitivity of SF. Practically, based on blood metabolites profiles obtained in TEMP, our biomarkers could be used to sort pigs according to their ability to cope with tropical conditions. Eventually, the application of such sorting is to enrich with more robust animals the pig population that is intended for export to warm regions. Additionally, the absence of correlation between the predicted sensitivity and the average back fat thickness in TROP suggest that a selection based on our model would not negatively impact fatness indicator. In addition, it would favor higher growth rates in TROP without negative impact on production traits in thermoneutral conditions. Finally, the reliability of our diagnostic method to improve the farm productivity needs to be validated in an independent and larger scale trial. To the best of our knowledge, no similar result has previously been shown in pigs.

In this study, we focused on identifying the best predictors of sensitivity. This does not provide evidence or causal link between the found predictors and the biological mechanisms that lead to sensitivity to heat stress. The biological entities that are used as predictors (here metabolome profiles) in machine learning based studies are not necessarily causally involved with the phenotype of interest [43]. However, although the aim of the current article was not to dissect the biological mechanisms of thermal tolerance, the highlighted metabolomic predictors in our study showed interesting interconnections within the metabolic network. Indeed, proline and arginine are both non-essential amino acids that derive from glutamate. Tian and a (2015), using 1HNMR and LC-MC approaches, showed that proline, arginine, ornithine and citruline among the most reliable plasma metabolomic biomarkers in lactating dairy cows [44]. Additionally, proline and glutamate can be precursors of citrulline biosynthesis, which has important hypothermic function, as shown in chicken [45]. Indeed, citrulline has been shown to increase endogenous nitric oxide production that can promote heat dissipation from skin [46]. However, higher proline levels in TEMP were associated to robustness and to lower the average rectal temperature in TROP. From these observations, we may suppose that robust SF would have a more active urea cycle which may prove more efficient for citrulline production and for thermoregulation in warm environment. In the metabolomic profiles, the buckets related to citrulline have not been identified yet. Further investigations on blood level of citrulline in TROP are needed to corroborate this hypothesis.

## Conclusion

In conclusion, firstly we propose a novel approach to estimate sensitivity to long term heat stress. Secondly, we showed that the plasma metabolomic profile is a promising tool to assess the sensitivity to heat stress from thermoneutral sample collections. Further studies coupling this metabolomic study with a genome wide association study could help highlighting differential mechanisms of adaptation to HS.

## Supporting information

S1 Data File Metabolomic and phenotypic datasets. (XLSX)

S1 Fig Monthly variations of THI. The figure shows variations of THI either in TEMP (red curve) or in TROP (blue curve) region during the trail that occurs between the years 2013 and 2014.
(TIF)

S2 Fig Principal variance component analysis. PVCA assessed the weighted average proportion of variance (WAPV) imputed to batch (B), sire family (SF), sex (S), and climate (C) factors on phenotypic or metabolomic dataset before adjustment (A and C respectively) and after adjustment (B and D, respectively).
(TIF)

S3 Fig Feature selection and validation method of *s*PLS-DA. The optimal numbers of buckets to select on the 3 components of sPLS-DA were chosen so as to minimize the balanced error rate of prediction assessed by cross validation (A). The sensitivity and specificity of *s*PLS-DA that was built on the number of selected variables were assessed with ROC curve (B).
(TIF)

S1 Table “t” dataset. Welsh “t” statistics for climate comparisons per SF on ADFI BW and RT.
(XLSX)

S2 Table Averaged animal performances and thermoregulation traits depending on the climate (C), the SF and the sex^1^. (XLSX)
