# Supplementary figures and images for: 1HNMR-Based metabolomic profiling method to develop plasma biomarkers for sensitivity to chronic heat stress in growing pigs
Source: PLoS One. 2017 Nov 27;12(11):e0188469. doi: 10.1371/journal.pone.0188469 (PMC5703499; doi:10.1371/journal.pone.0188469)

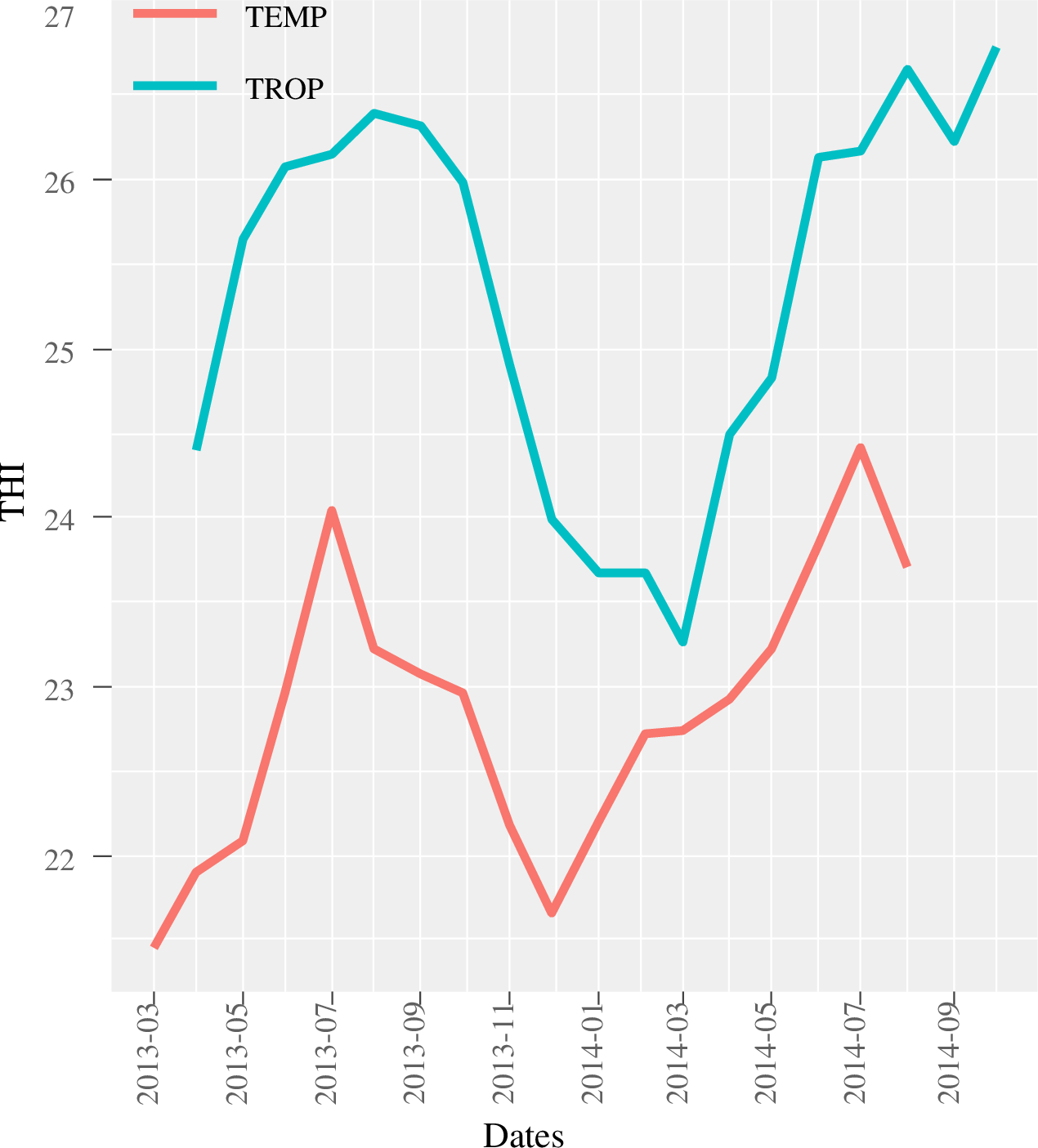

Supplement: S1 Fig — The figure shows variations of THI either in TEMP (red curve) or in TROP (blue curve) region during the trail that occurs between the years 2013 and 2014. (TIF) [file pone.0188469.s002.tif]

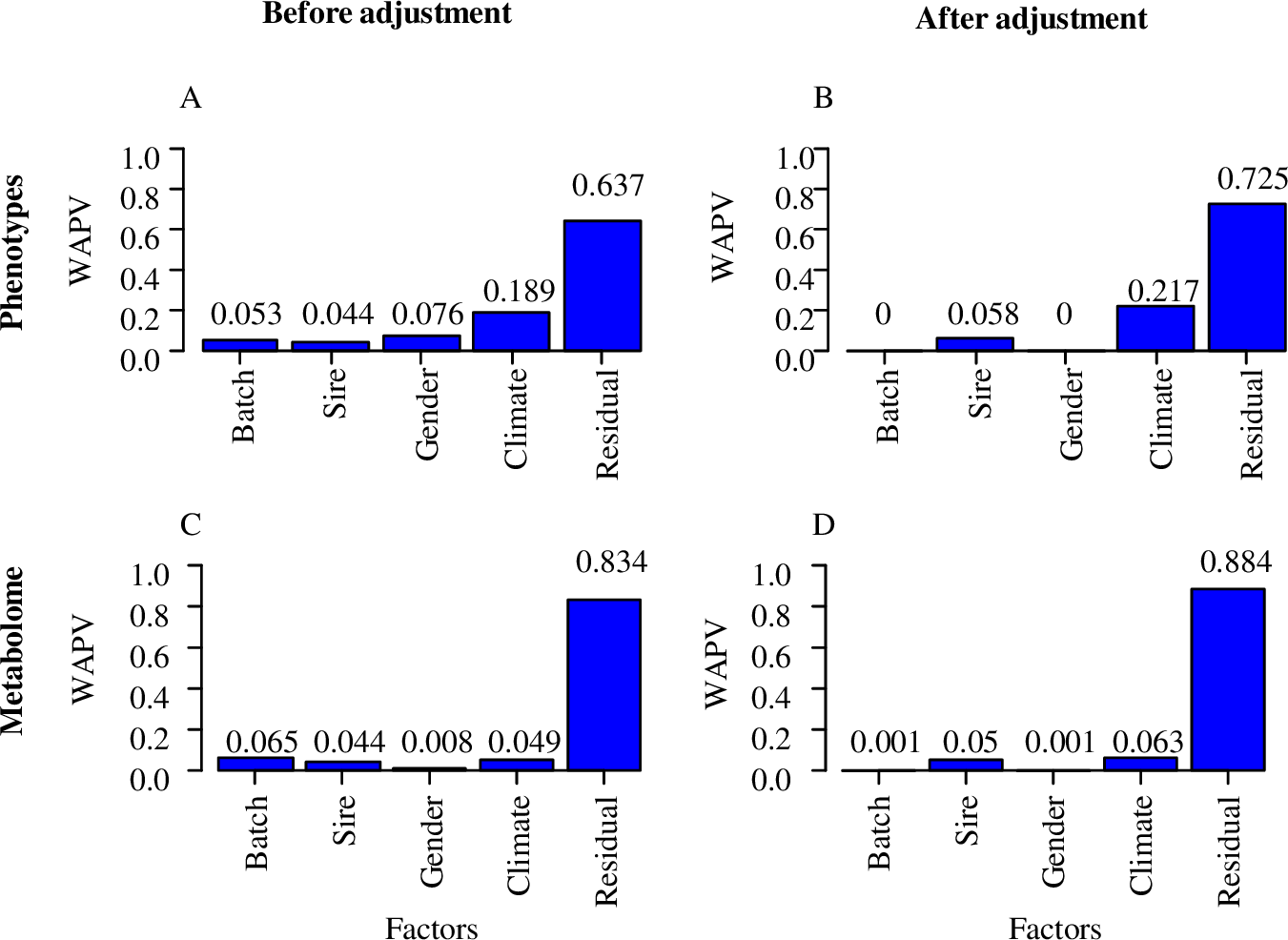

Supplement: S2 Fig — PVCA assessed the weighted average proportion of variance (WAPV) imputed to batch (B), sire family (SF), sex (S), and climate (C) factors on phenotypic or metabolomic dataset before adjustment (A and C respectively) and after adjustment (B and D, respectively). (TIF) [file pone.0188469.s003.tif]

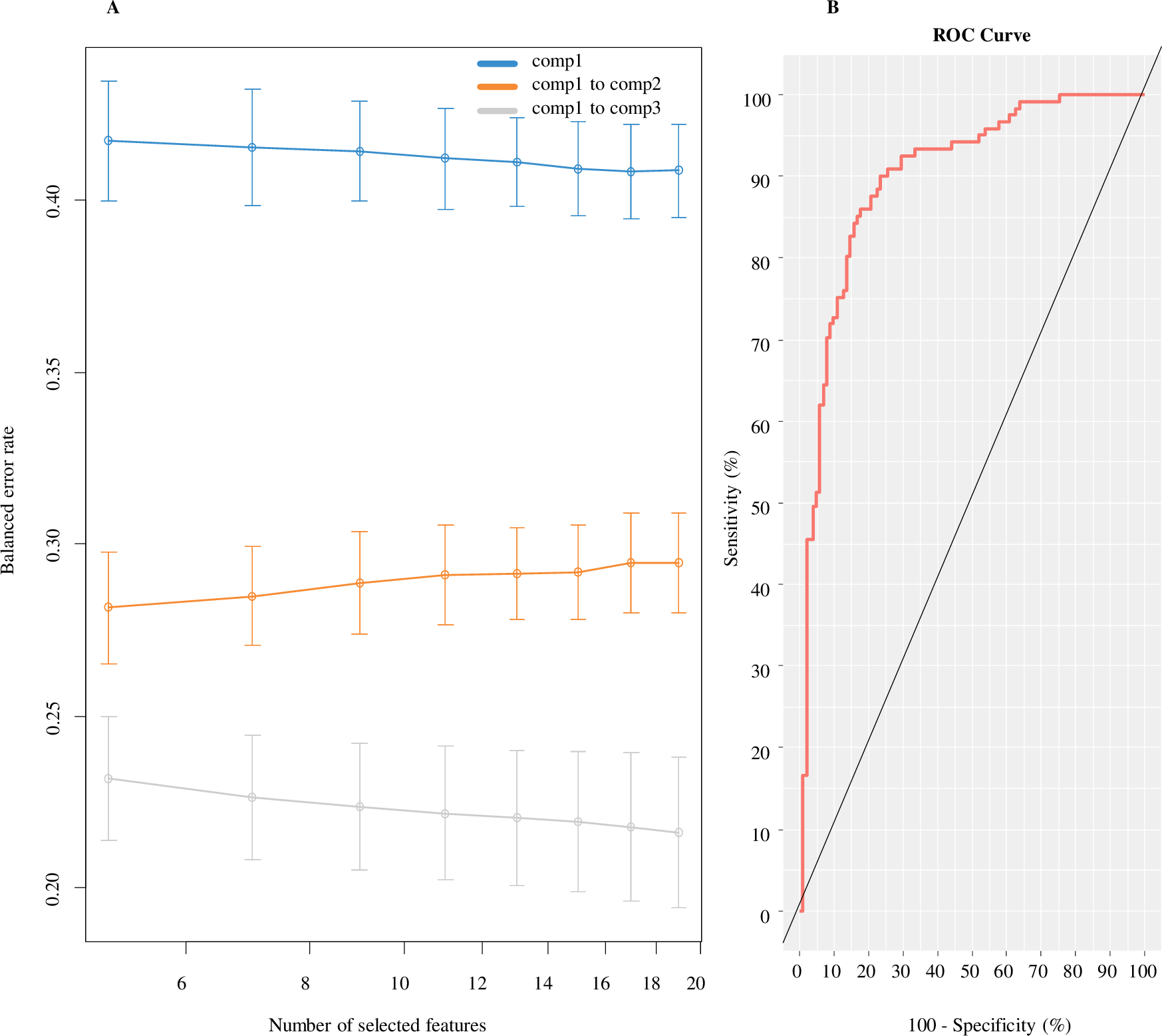

Supplement: S3 Fig — The optimal numbers of buckets to select on the 3 components of sPLS-DA were chosen so as to minimize the balanced error rate of prediction assessed by cross validation (A). The sensitivity and specificity of sPLS-DA that was built on the number of selected variables were assessed with ROC curve (B). (TIF) [file pone.0188469.s004.tif]
